# Teleworking While Sick: A Three-Wave Study of Psychosocial Safety Climate, Psychological Demands, and Presenteeism

**DOI:** 10.3389/fpsyg.2021.734245

**Published:** 2021-10-28

**Authors:** Caroline Biron, Maria Karanika-Murray, Hans Ivers, Sandra Salvoni, Claude Fernet

**Affiliations:** ^1^Department of Management, Université Laval, Québec, QC, Canada; ^2^Center of Research for Sustainable Health–VITAM, Québec, QC, Canada; ^3^Centre of Expertise for the Management of Occupational Health and Safety, Québec, QC, Canada; ^4^Department of Psychology, School of Social Sciences, Nottingham Trent University, Nottingham, United Kingdom; ^5^School of Psychology, Université Laval, Québec, QC, Canada; ^6^Department of Human Resources Management, Université du Québec à Trois-Rivières, Trois-Rivières, QC, Canada

**Keywords:** COVID-19, pandemic, telework, psychosocial safety climate (PSC), psychological demands, presenteeism

## Abstract

**Introduction:** The COVID-19 pandemic has led to a significant increase in the proportion of employees for whom teleworking became mandatory. Presenteeism, or the behavior of working while ill, has hardly been studied in the context of telework. The pandemic forced millions of workers to abruptly transition to working from home for a prolonged period of time, leaving employers often unaware of their health status or work capacity of the workers. This change also eroded the work experience itself, the workplace, and their protective impact on both individual health and work outcomes. This study focused on the longitudinal relationships among psychosocial safety climate (PSC), a lead indicator of workplace conditions, psychological demands, an indicator of quality of work, and presenteeism among a representative sample of teleworkers. PSC was expected to have an indirect impact on presenteeism with psychological demands as a mediator of this impact.

**Method:** We collected the data from a representative sample of teleworkers in the first months (T1: April, T2: June, and T3: December 2020) of the pandemic using a three-wave online survey (*n* = 275). We tested a model of PSC as a determinant of presenteeism in teleworkers with psychological demands as a mediator. A cross-lagged panel model was estimated to test cross-sectional and longitudinal relationships.

**Findings:** As expected, psychological demands increased over time. Contrary to expectations, the prevalence of presenteeism remained unchanged while PSC increased over time. The data fully supported the mediating effect of psychological demands such that a higher evaluation of PSC at T1 led to lower psychological demands at T2, which led to reduced presenteeism at T3. We also found a reciprocal relationship, with higher psychological demands at T2 leading to decreased evaluation of PSC at T3. These results show that the perception of teleworkers on their organization as giving a high priority to their psychological health is an important determinant of their work experience, ultimately influencing their decision to work while ill. The context of the pandemic has highlighted the importance of a positive workplace climate and working conditions for reducing the behaviors that can be harmful to health and productivity. Implications for theory and practice, beyond the pandemic, are discussed.

## Introduction

The possibility to work from home used to be considered as a privilege only available to a few, but the COVID-19 pandemic has created a shift in this work arrangement and forced workers and employees (hereafter both referred to under the umbrella term “workers”) from a wide range of occupations and employment sector to work primarily or fully from home (Kramer and Kramer, 2020). The proportion of workers who predominantly work from home varies depending on the context and nature of work, but it increased dramatically in several countries during the pandemic. For example, in Canada, by early 2021, 32% of Canadian employees aged 15–69 were working most of their hours from home, compared to only 4% in 2016 (Mehdi and Morissette, 2021). In a sample of managerial and professional workers across 29 European countries, Ipsen et al. (2021) found that 84.1% worked exclusively from home during the pandemic. The proportion of EU-27 employees who usually worked from home hardly increased between 2006 (4.6%) and 2019 (5.4%) (Samek Lodovici, 2021). In contrast, during the pandemic, 34% of the workforce worked from home full-time across all sectors and occupations (Eurofound, 2020).

In the Canadian province of Québec, the government imposed general lockdown in March 2020, by closing all but essential shops such as groceries and pharmacies to reduce the transmission of the virus. In June 2020, telework was favored and recommended by the government, but in December 2020 it became compulsory for all employees of all sectors who carry out administrative or office work, except for workers whose physical presence is essential for the continuation of the business. National data from Statistics Canada show that 37% of the companies in Québec reported that teleworking was possible for all employees. In August 2020, 27% of these companies reported that all their employees were teleworking while 14% expected their entire workforce would continue to primarily telework after the pandemic (Institut de la statistique du Québec, 2021). This contrasts sharply with the data from 2006 showing that only 5% of Quebec employees actually worked primarily from home (Gagnon, 2009).

A change of this magnitude raises several important questions. One concern relates to the psychological demands perceived or experiences by teleworkers. Unhealthy levels of screen time, more time spent at online meetings, work during evenings and weekends, higher pressure to produce, and no respect for time and boundaries are just a few examples of the impacts of the pandemic on teleworkers highlighted by Moss (2021). In a survey of over 1,500 employees in several employment sectors in 45 countries in the autumn of 2020, Moss (2021) found that 89% of the workers reported a decrease in their work-related well-being, with an increase in workload being the strongest explanatory factor for this trend. The survey of Statistics Canada of the active population revealed that 35% of all “new teleworkers” (those who began working primarily from home due to the pandemic) reported working more hours per day than previously, whereas only 3% reported fewer working hours (Mehdi and Morissette, 2021). Nearly half (48%) of the teleworkers indicated that they worked for longer hours (Mehdi and Morissette, 2021). Teleworkers tend to work more hours and more intensively than employees working onsite (Messenger et al., 2017; Tavares, 2017). Drawing on this research, in this study, we focus on psychological demands, which refer to the amount of work, mental demands, and time constraints experienced by workers (Karasek et al., 1998). The question arises whether this increase in psychological demands can be prevented in a context where the management of an organization show that they value the psychological health and well-being of workers—in other words, in the context of a high psychosocial safety climate (PSC; Dollard and Bakker, 2010). In a high PSC context where the psychological health of workers is a priority, managers are aware of the negative effects of high job demands, and ensure that there are policies, practices, and procedures to protect employees from harmful work conditions (Idris et al., 2011).

A second question raised by this sudden and dramatic increase in the number of teleworkers relates to their experiences of PSC in their organizations, especially due to the physical and often social isolation from their workplace, colleagues (Tavares, 2017), and line managers (Contreras et al., 2020). In an economic context characterized by strong pressures on organizations, do the economic imperatives take precedence over the concern and priority given to the psychological health of workers (Dollard et al., 2019)? Can a concern for work-related well-being be communicated well enough considering the remote nature of the work and ensuring that teleworkers feel safe to express any difficulties to their colleagues and line managers? While mental health has broadly deteriorated in the general population due to the COVID-19 crisis (Salari et al., 2020), the mental health of those who are primarily teleworking remains relatively unexplored. An international study by Ipsen et al. (2021) conducted during the early stages of lockdown concluded that, although individuals experienced teleworking more positively than negatively, nearly half also experienced deteriorating mental health. This raises questions about the ability of employers to prioritize the well-being of teleworkers and to remain accessible to those in difficulty and the strategies that can enable those workers to share and resolve issues and access appropriate support.

A third question raised by this change in the proportion of teleworkers and the resulting increase in psychological demands and in mental health problems is presenteeism. Presenteeism has been defined as being physically present at work despite illness (see Karanika-Murray and Cooper, 2018; Ruhle et al., 2019 for an overview). In the context of telework, we adopt the definition of presenteeism as the state of attending work when one is unwell (Karanika-Murray and Cooper, 2018) or the act of working in a state of ill-health (Ruhle et al., 2019). Presenteeism has been linked to negative health conditions, be it physical or psychological (Johns, 2010; Gosselin and Lauzier, 2011), with the latter being the most prevalent (Klachefsky, 2013). Presenteeism is highly prevalent across all occupations and sectors (Karanika-Murray and Cooper, 2018). However, a very few studies have investigated presenteeism in teleworkers. One rare study showed that teleworking is linked to increased presenteeism through lifting any barriers to overworking (Steidelmüller et al., 2020). Indeed, data from the sixth wave of the European Working Conditions Survey 2015 indicated a strong positive association between teleworking and presenteeism, especially for those who work from home several times a week or daily (Steidelmüller et al., 2020).

Providing answers to these questions is essential for informing discussions about the viability of telework following the COVID-19 pandemic. Among the many emerging changes in working practices brought about by the pandemic (Kniffin et al., 2021), telework is becoming a more and more widespread practice that needs to be better understood in terms of its impact on health issues. Considering that telework is likely to become a permanent solution for many organizations in the future, there is a need to better understand how it can become a healthy and productive arrangement for both employers and workers. Thus, the main thrust of this paper is to investigate the reality of teleworkers who primarily worked from home throughout the pandemic. The findings will help to understand how contextual factors (PSC) and proximal job factors (psychological demands) together shape the presenteeism behavior, with potential implications for sustainable organizational interventions that can be developed to improve the health and well-being of teleworkers.

### Teleworking During the Pandemic

Research within the current field of teleworking is limited in two ways. A first and methodological limitation is that most studies investigating teleworkers have been conducted with homogeneous groups such as the self-employed, knowledge workers, or high-skilled workers (such as professionals and managers). A very few studies have investigated this heterogeneous group of workers who are teleworking most of the time and without a choice in the matter. A second limitation concerns the theoretical implications of PSC for presenteeism in the context of telework. PSC highlights the importance of protecting employees from poor quality of work by providing sufficient and adequate resources, such as autonomy, supervisor support, and healthy relationships, which can mitigate the negative effects of high job demands (Law et al., 2011). However, PSC has hardly been investigated in teleworkers and is therefore unknown if it can protect this group from otherwise poor working conditions.

Before proceeding, it is important to define teleworking. A few studies vary in terms of the definition and measurement of teleworking as there are several nuances in the terms that are used to characterize those who work away from their workplaces (e.g., virtual teams, remote work, telecommuting, and teleworking) (Allen et al., 2015). We define home-based telework, hereafter referred to as telework, as: “work performed by those whose remote work is from the home” (Allen et al., 2015, p. 43; also following Steidelmüller et al., 2020).

It is also important to distinguish between the flexibility available to employees to work from home, on one hand, from the type of mandatory or forced telework that was provoked by the pandemic, on the other hand. The former was a trend strongly on the rise before the pandemic with, for example, 20% of the US employers offering this option in 1996, compared to 60% in 2016 (Society of Human Resource Management., 2016). Working from home as a flexible working arrangement is very different as an experience form being “forced” into it, often without the appropriate tools, support, or management systems to accommodate large groups of workers away from the organizational premises or often without clear guidelines or performance expectations. Ipsen et al. (2021) found that, despite some positive aspects, 45% workers experienced teleworking as a mostly negative experience, citing as main disadvantages missing their colleagues, poor physical work conditions in the home office, and feeling isolated at home.

Among the work issues and outcomes most frequently identified with telework are disruption of work-life boundaries, overwork, presenteeism, social isolation, barriers to career progression or promotion, and the lack of support (Montreuil and Lippel, 2003; Tavares, 2017; Ipsen et al., 2021). Teleworking can also have negative impacts on well-being due to potential overcommitment, overwork, and the lack of time to recuperate when boundaries between work and personal life are unclear (Grant et al., 2013). For presentees, teleworking is an ally as it allows them to more easily adjust their work pace according to their health status, take breaks, and do fewer or less demanding tasks, potentially though masking the seriousness of a health condition. However, such adjustment latitude is less likely when job demands are high (Johansson et al., 2015), which makes presenteeism more likely under high demands. Despite hopes that telework would lighten work schedules because of the time saved in travel, recent data suggest that the time spent working has increased among teleworkers (Lundberg and Lindfors, 2002; Peters et al., 2008; Tavares, 2017).

By allowing for more work scheduling flexibility, telework has enabled workers to connect remotely, even when they are feeling less well, and even take time off due to their health conditions and without mentioning it to their line managers. The extent of presenteeism in the context of telework may thus be underestimated. Steidelmüller et al. (2020) highlight the three main reasons for an increased risk of presenteeism among teleworkers: because they do not have to travel to work and are in a more convenient work environment, they do not risk contaminating their colleagues in case of a contagious disease, and finally, they are not under the supervision of their managers nor are they visible to colleagues and do not have to justify working when they should not. Thus, they have fewer barriers to work even when they are unwell: “In the worst case of sickness I said: Okay, I do not come to the office, I'll stay at home. Then, I just worked out of the bed” (Ruhle and Süß, 2020, p. 248). This quote illustrates the issues surrounding presenteeism in the telework context. Given the lack of clear boundaries between home and work and the difficulty for teleworkers to efficiently manage these boundaries (Ashforth et al., 2000; Kniffin et al., 2021), we expect that psychological demands will increase over time. When teleworking is mandatory, employees may also experience difficulties in being efficient at work due to the normative demands that stem from personal life and occur during work time (e.g., family responsibilities and emergencies such as homeschooling during lockdown).

Based on these challenges experienced by teleworkers and in line with the lack of recovery spiral (Hobfoll, 2002), we expect that presenteeism will increase over time. Whereas teleworkers devote considerable energy to meet work requirements, the resources available to sustain such an effort over time are limited (Hobfoll, 2002) and may result in loss of psychological resources and in other negative outcomes including presenteeism. Therefore, we can expect that, over time, psychological demands and presenteeism of teleworkers will both increase.

*H1: Perception of psychological demands among teleworkers will increase over time*.
*H2: Presenteeism prevalence among teleworkers will increase over time*.

### Psychosocial Safety Climate

Excessive psychological demands can influence the decision of an employee to work despite illness (Miraglia and Johns, 2016) and therefore it is important to examine the factors that can protect presentees. PSC has been shown to influence working conditions, including both job demands and job resources (Hall et al., 2010; Idris and Dollard, 2011; Idris et al., 2011; Law et al., 2011; Dollard et al., 2012). As such, PSC is a leading indicator of work quality.

Considered to be a precursor of unsafe work conditions and psychosocial risk factors, PSC refers to the perceptions of employees regarding the policies, practices, and procedures for the protection of worker psychological health and safety (Dollard and Bakker, 2010). PSC emphasizes the important influence of organizational climate on job design and psychological health (Dollard et al., 2012). Four organizational domains define PSC: senior management commitment to stress prevention, senior management priority for psychological health vs. productivity imperatives, organizational participation and involvement in managing psychological health risks, and organizational communication about psychological health issues (Dollard et al., 2019).

Psychosocial safety climate has been shown to be an important organizational resource (Garrick et al., 2014) that influences the work experience of one, including their job demands and job resources (Dollard and Bakker, 2010). High levels of PSC have been linked to the decreases in experienced job demands and load, in both cross-sectional (Dollard and Bakker, 2010) and longitudinal research (Idris et al., 2014). In a study by Idris et al. (2014), it has been suggested that the period required for macro-level contextual variables to influence work design and organization is rather short, 3 months in their study. Although PSC has not been explored in the context of telework, it is expected that PSC will be an important resource for teleworkers, especially because of the remote nature of work and distance from managerial and collegial support during the pandemic, where important immediate resources are not available or their availability decreases over time. Therefore, we can expect that over time, in the context of the pandemic, the perceived PSC of teleworkers would decrease because of the loss of social and otherwise contact with work, and that higher perceived PSC would also support reduced experience of psychological demands.

*H3: PSC perceptions among teleworkers will decrease over time*.
*H4: PSC will have a negative effect on teleworkers' psychological demands over time*.

Indeed, job demands are an important causal factor for presenteeism (Demerouti et al., 2009). Research suggests that presenteeism is more sensitive to job demands than absenteeism. For example, regular overtime has been found to decrease absenteeism but to increase presenteeism (Böckerman and Laukkanen, 2009). Similarly, meta-analytic estimates have revealed stronger links between job demands and presenteeism (*r* = 0.24) compared to job demands and absenteeism (*r* = 0.05) (Miraglia and Johns, 2016).

In agreement with the Job Demands-Resources (JD-R) model (Bakker and Demerouti, 2017), job demands vary in nature and include cognitive, physical, emotional, and psychological demands. Psychological demands, such as work pace, time pressures, and high workload, have been found to be strong predictors of presenteeism (Claes, 2011; Deery et al., 2014; Baeriswyl et al., 2017). Data from the fifth European survey on working conditions (2010) suggest that time demands (working outside work hours such as at weekends and during non-work time) are strongly linked to presenteeism (Nordenmark et al., 2019). Other cross-sectional (Janssens et al., 2016; McGregor et al., 2016) and longitudinal studies (Demerouti et al., 2009; Oshio et al., 2017) have shown that the demands of high workload and time pressures predict the prevalence of presenteeism.

*H5: Over time, psychological demands will be associated with increased presenteeism*.

Recently, direct relationships between PSC and presenteeism have been supported (Liu et al., 2020). However, the workplace climate also shapes the working conditions that in turn shape the work experience of one (Dollard et al., 2019). The effects of PSC on mental health outcomes *via* working conditions have been supported in longitudinal studies (Idris et al., 2014; Dollard et al., 2017; Huyghebaert et al., 2018; Loh et al., 2018). For example, Dollard et al. (2012) found that an experienced PSC among one group of nurses in one work unit predicted the ratings of workload, job control, supervisor social support, and psychological strain in a different group of nurses from the same work unit, over 24 months. Dollard et al. (2012) also found that the effects of PSC on psychological strain were through psychological demands. Indeed, the lack of resources can make one more vulnerable to resource loss and less capable of resource gain (Conservation of Resources Theory, COR; Hobfoll, 1989; Freedy and Hobfoll, 2017), which implies that a weak PSC may increase the perceptions of psychological demands. Because PSC and psychological demands are expected to have a direct impact on presenteeism, we expect that psychological demands will mediate the relationship between PSC and presenteeism. This is consistent with the health impairment process of the JD-R model (Bakker and Demerouti, 2017) whereby a poorly designed job (as it may be reflected by low PSC) can increase job demands and in turn lead to strain and health problems.

*H6: Psychological demands mediate the relationship between PSC and presenteeism, such that positive perceptions of PSC will lead to lower psychological demands that will in turn lead to lower presenteeism prevalence*.

## Methods

### Designs and Procedures

This was a longitudinal cohort study that used the data collected between April 2020 and December 2020 during the COVID-19 pandemic and following the pandemic-related restrictions in Canada. The first data collection wave was in April 2020, during the strict lockdown the Québec province, where Public Health issued an incentive to all employers to encourage telecommuting for all employees, as permitted by the nature of the work. The second data collection wave was 3 months later, at the end of June 2020 where most stores and shops had opened, and schools had reopened, and the summer holidays were about to start. Except for key workers and those necessary for the pursuit of essential organizational activities, telework was still highly recommended by the Québec government. The third data collection wave was at the end of November and early December 2020, about 3 months after the summer holidays (July–August). This was the onset of the second wave of COVID-19 when lockdown measures came back into effect. Stores were closing, and teleworking was therefore mandatory for people working in offices from December 17, 2020 to January 10, 2021. Data were collected *via* a web panel representative of the Quebec population. Web panels are increasingly recommended for population-based studies (Svensson, 2014). A random prize draw was offered at each time point, with an increasingly higher prize for those who participated in more than one wave.

### Participants

The web panel included 60,000 adults (2020 population of Québec of those aged 18–64 comprises 5.3 million). A total of 6,000 were invited randomly, of whom 1,450 replied that they had worked over the past 7 days and agreed to participate. The sample consisted of 553 participants working from home at least 80% of their work hours (the rationale for the 80% cut-off is provided below), of whom 275 were also teleworking at each time wave, had complete data on independent variables at T1, and had completed at least two of the three waves. These participants (*n* = 275) were considered to be teleworkers and were included in the analyses.

### Measures

#### Demographic Variables

Demographic variables demographic information included gender, age, occupation, employment sector, type of work contract, and education (see Table 1). Employment sector classification was based on the North American Industry Classification System (Statistics Canada., 2002), which was used by the last population-based study conducted on the working population of Québec (Vézina et al., 2011).

**Table 1:** Description of the sample (*n* = 275, unweighted, at T1).

**Socio-demographics**	***n*** **(%)**
**Gender**	
Male	117 (42.6)
Female	158 (57.4)
**Age**	
20–34	39 (14.2)
35–54	178 (64.7)
55+	58 (21.1)
**Occupation**	
Top/middle manager	25 (9.1)
Line manager	24 (8.8)
Professional	142 (51.8)
Clerical/admin	41 (15)
Technical	42 (15.3)
Blue-collar	0
**Employment sector**	
Primary/construction	12 (4.4)
Manufacturing	10 (3.6)
Services (information, arts, leisure, hospitality, retail)	34 (12.4)
Health/social aid	18 (6.6)
Education	30 (10.9)
Public/governmental	124 (45.1)
Finances/insurance	36 (13.1)
Others	10 (4)
**Contract**	
Full time	219 (79.6)
**Education**	
Highschool or less	13 (4.7)
College	76 (27.6)
University	186 (67.6)

#### Teleworking

Teleworking using an open-ended response format, respondents were asked to indicate the number of hours they had worked from home and the number of hours worked on site in the past week (7 days) and normally (before the onset of the COVID-19 pandemic). We considered teleworkers to be the ones who reported working from home at least 80% of their work hours at each of the three data collection waves. The rationale for the 80% cut-off was based on the average number of hours of teleworking (*M* = 29.3 h over the past week at T1), considering that a typical employment contract of Québec's is 37 h (29.3/37 *h* = 79%).

#### Presenteeism and Absenteeism

Presenteeism and absenteeism presenteeism was measured using an open-ended response format where respondents indicated how many days they had worked while they had been unwell over the past 7 days: “in the last week (7 days), how many days did you work while you had a health problem?” A definition of health problems was provided as “any physical or emotional problem or symptom.” For absenteeism, the same format was used but the focus was on the number of work hours missed, including being late or having to leave early, because of the health issue. Although a 12-month recall period is often used in presenteeism studies, we used a shorter period to reduce recall bias according to Navarro et al. (2019) and Ruhle et al. (2019).

#### Psychological Demands

Psychological demands this construct covers the quantity of work, mental requirements, and time constraints at work. The six items forming this scale were based on the short version of the Job Content Questionnaire (Karasek et al., 1998). The French version of the scale has been shown to have acceptable psychometric properties (Brisson et al., 1998). Following a CFA analysis, only four of the original six items were used. Item 4, which was referred to contradictory demands, and item 6, which was referred to as being often interrupted at work, had low loadings between 0.30 and 0.50 and were removed. The response scale ranged from 1 (strongly disagree) to 4 (strongly agree). An example item is: “My job requires working very fast.” Internal consistency (α = 0.77) and composite reliability (T1 = 0.807, T2 = 0.795, and T3 = 0.795) were within acceptable levels.

#### Psychosocial Safety Climate

Psychosocial safety climate (PSC) was measured using PSC-4 (Dollard, 2019). Items refer to the perception of respondents on the priority given to mental health issues by the top management of their organization, the commitment of top management, and the participation and communication from all levels of the hierarchy to prevent mental health problems at work. The responses scale ranged from 0 (strongly disagree) to 5 (strongly agree), such that a higher score implies a climate perceived as more favorable. An example item is: “senior management shows support for stress prevention through involvement and commitment.” Internal consistency (α = 0.94) and composite reliability (T1 = 0.936, T2 = 0.935, and T3 = 0.918) were high.

### Analyses

Post-stratification weights were computed to ascertain the representativeness of the adult population of Québec according to gender, age, rural/urban area, education, and language. To evaluate whether mean levels of the dependent (presenteeism) and independent variables (PSC and psychological demands) changed over time, a series of random intercept mixed models with time (three waves) as a fixed effect were performed. A cross-lagged panel model (three waves) was estimated to test cross-sectional and longitudinal relationships between PSC, psychological demands, and presenteeism. A longitudinal mediation effect was tested according to the guidance by Taris and Kompier (2006) to include the three waves of data. This is recommended to enable the estimation of the directional associations between PSC at T1 and psychological demands at T2 (alpha relation), and psychological demands at T2 and presenteeism at T3 (beta relation), while controlling for autoregressive effects (correlations between each consecutive measurement for each variable). Model measurement fit and composite reliability were estimated. Model invariance according to demographic variables (gender, age group, education, and having children younger than 18 years of age) was tested using chi-squared difference (Satorra and Bentler, 2010). Analyses used longitudinal weights and were performed with SAS 9.4 and MPlus 7 using standard two-tailed 5% alpha.

## Results

### Non-response Analysis

As mentioned earlier, of the 553 participants who teleworked at least 80% of the time, we used a subsample of 275 who had no missing values on the independent variables at T1 and who had completed at least two measurement waves. We verified the differences between dropouts and our sample (*n* = 275) of teleworkers. No differences were detected for gender [χ^2^ = 0.23 (1), *p* = 0.64] and age group [χ^2^ = 55.2 (1), *p* = 0.76]. However, differences were found for occupation [χ^2^ = 29.0 (5), *p* = 0.00]. No differences were found for managers (*p* = 0.93), professionals (*p* = 0.37), clerical workers (*p* = 0.16), and technicians (*p* = 0.70), but there was a significant difference for unskilled workers [χ^2^ = 21.3 (1), *p* = 0.00]. Our panel group includes 0% of unskilled workers, but the comparative (*n* = 553) group included 8% of unskilled workers who spend 80% of their time teleworking. Differences were found according to the employment sector [χ^2^ = 33.37 (4), *p* = 0.00], but 25% of the cells had fewer than five participants. Overall, our study sample included a higher number of participants from the public sector (49 vs. 36%) and the finance/insurance sector (22 vs. 6%) but fewer from retail (5 vs. 24%) compared to the dropouts. Our sample also included a higher number of participants who worked full-time (80%), whereas the dropout group included only 68% of full-time employees [χ^2^ = 10.65 (1), *p* = 0.00]. Finally, our panel is comprised of a higher proportion of workers with a university degree (63%) compared to the dropout group (57.8%) and a lower proportion of workers with a high school degree (9 vs. 17%) [χ^2^ = 8.08 (2), *p* = 0.02].

Considering that no gender and age differences were found and that the very nature of telework implies that some professionals are unable to work from home, the differences between our panel and the dropouts were to be expected given the nature of telework. We concluded that there were no issues related to panel loss.

### Descriptive Statistics

Descriptive statistics for the demographic variables at T1 are displayed in Table 1. The correlations between variables at each time are described in Table 2.

**Table 2:** Correlations (*r*) for the study variables (T1: April 2020, T2: June 2020, and T3: December 2020) (*n* = 275).

	**Psychosocial** **safety** **climate T1**	**Psychological** **demands** **T1**	**Presenteeism** **T1**	**Psychosocial** **safety** **climate T2**	**Psychological** **demands T2**	**Presenteeism** **T2**	**Psychosocial** **safety** **climate T3**	**Psychological demands** **T3**	**Presenteeism** **T3**
Psychosocial safety climate T1	1.00								
Psychological demands T1	−0.19^***^	1.00							
Presenteeism T1	−0.04	0.17^***^	1.00						
Psychosocial safety climate T2	0.67^***^	−0.12^*^	−0.07	1.00					
Psychological demands T2	−0.23^***^	0.73^***^	0.12^**^	−0.29^***^	1.00				
Presenteeism T2	−0.09	0.08	0.48^***^	−0.28^***^	0.16^**^	1.00			
Psychosocial safety climate T3	0.58^***^	−0.26^***^	−0.06	0.74^***^	−0.29^***^	−0.25^**^	1.00		
Psychological demands T3	−0.06	0.61^***^	0.16^*^	−0.04	0.64^***^	0.07	−0.26^***^	1.00	
Presenteeism T3	−0.08	0.18^*^	0.47^***^	−0.13	0.24^**^	0.49^***^	−0.19^*^	0.30^***^	1.00

* *p < 0.05*,

** *p < 0.01*,

*** *p < 0.001*.

Table 3 shows the means for all variables at each time. Psychological demands showed an increase over time (*F* = 32.40, *p* < 0.00) and were significantly higher at each consecutive time: T1 = 2.32, T2 = 2.50, and T3 = 2.57, thus supporting H1. Contrary to H2, the number of days of presenteeism remained stable across measurement times (T1 *M* = 1.28, T2 *M* = 1.29, T3 *M* = 1.06, *F* = 1.18, *p* = 0.31; overall *M* = 1.21 days of presenteeism in the past week). In contrast, absenteeism decreased from T1 (*M* = 0.21) to the other two waves (T2 *M* = 0.11, T3 *M* = 0.10, *F* = 3.60, *p* = 0.03). In terms of frequency of presenteeism in the past week, at T1, 61.8% reported no presenteeism, 11% worked while unwell at least 1 day, whereas 27.8% worked while unwell at least 2 days during the preceding week. In contrast, at T1, 88.8% reported no absenteeism, 6.9% were absent between 1 h and 1 full day, and 4.3% were absent for 2 days or more. These findings are reported for a comparison but considering the distinct etiology of absenteeism and presenteeism (Miraglia and Johns, 2016), only presenteeism was used in the subsequent analyses.

**Table 3:** Means (M), test of mean differences, SDs, for the study variables (T1, T2, and T3, *n* = 275), reliability coefficients (Cronbach's α at T1).

	**T1 (April 2020) *M*(SD)**	**T2 (June 2020) *M*(SD)**	**T3 (December 2020) *M*(SD)**	***F*** **(2,429)**	**α**
1. Psychosocial safety climate (scale 0–5)	3.71^a^ (0.06)	3.64^a^ (0.06)	3.83^b^ (0.07)	5.33 (*p* = 0.005)	0.94
2. Psychological demands (scale 0–4)	2.32^a^ (0.03)	2.50^b^ (0.03)	2.57^c^ (0.04)	32.40 (*p* < 0.0001)	0.77
3. # days of presenteeism (scale 0–7)	1.28 (0.12)	1.29 (0.12)	1.06 (0.15)	1.18 (*p* = 0.31)	–

*Cronbach α measured at T1. Different subscripts (a,b,c) refer to differences in means between times of measurement at p <0.05*.

The data did not support H3. Contrary to H3, PSC increased over time (*F* = 5.33, *p* = 0.00) and was higher at T3 (*M* = 3.83) compared to T1 (*M* = 3.71) and T2 (*M* = 3.64).

### Cross-Lagged Panel Model

A CFA analysis was conducted to assess the measurement model by combining all times of measurement. Following previous recommendations (Little et al., 2007), each variable at Time 1 was also allowed to covary with its corresponding variable at Time 2 and Time 3. The results showed a satisfactory fit: χ^2^(df) = 641.88 (313), standardized root mean square residual (SRMR) = 0.081, comparative fit index (CFI) = 0.922, Tucker-Lewis index (TLI) = 0.912, and root mean square error of approximation (RMSEA) = 0.062.

**Figure 1:**
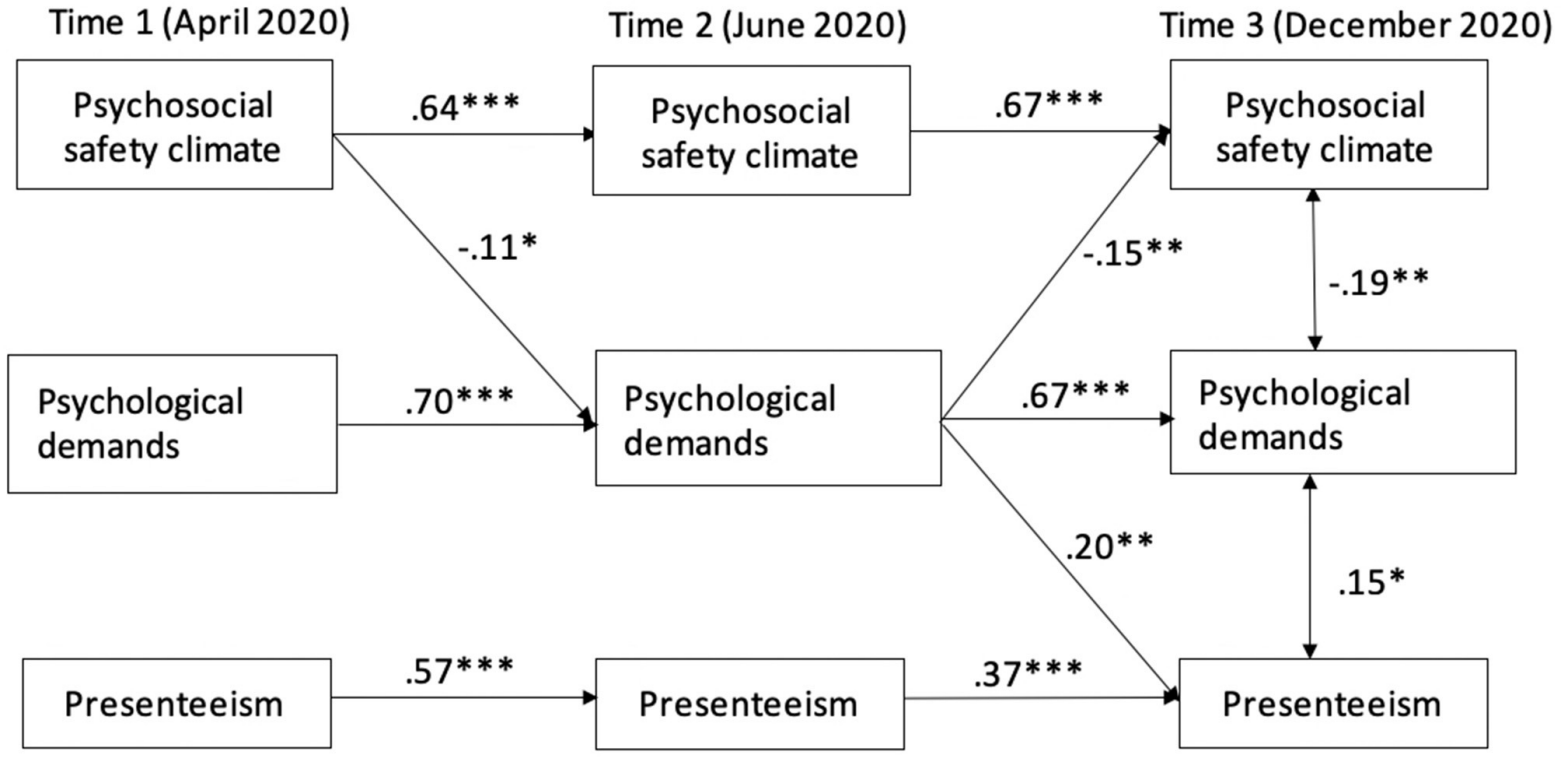
Three-wave mediation model of psychosocial safety climate and presenteeism for teleworkers (*n* = 275). *p < 0.05, **p < 0.01, ***p < 0.001.

Figure 1 illustrates the cross-lagged model for teleworkers, which showed a satisfying fit to the data with χ^2^ (12) = 33.90, *p* = 0.00, SRMR = 0.039, CFI = 0.964, TLI = 0.90, and RMSEA = 0.081.

The examination of standardized cross-path regression coefficients showed that PSC at T1 predicted increased psychological demands at T2 (*B* = −0.11, *p* = 0.04), confirming the alpha indirect relation (H4: independent variable → mediator). Psychological demands at T2 predicted an increased number of days of presenteeism at T3 (*B* = 0.20, *p* = 0.01), thus supporting the beta relation (H5: mediator → dependent variable). These two associations supported H6 on the presence of a longitudinal indirect effect of psychological demands on the relation between PSC and presenteeism (*B* = −0.021, *p* = 0.05). Cross-sectional associations between the three variables at T3 provided additional support for the indirect relationship (H6). Specifically, PSC was negatively correlated with psychological demands (*B* = −0.19, *p* =0.01), and psychological demands were positively associated with the number of days of presenteeism (*B* = 0.15, *p* = 0.04), but no significant association was found between PSC at T3 and presenteeism at T3 (*B* = −0.11, *p* = 0.13). Finally, a reciprocal longitudinal relationship was found between psychological demands at T2 and PSC at T3 (*B* = −0.15, *p* = 0.01).

Model invariance according to gender, age, education, and having children under 18 years old was also tested. We found no significant difference in the model across gender (*p* = 0.72), age groups (*p* = 0.38), or having children under 18 years old (*p* = 0.70). A significant difference was found for education [χ^2^ (33) = 61.20, *p* = 0.00] suggesting that the model had a lower fit for teleworkers without a university degree.

## Discussion

Using a three-wave cross-lagged panel design in a large, representative population sample, our data on teleworkers who primarily worked from home throughout the first 10 months of the COVID-19 pandemic showed a constant increase in psychological demands and an unchanged prevalence of presenteeism. Yet, teleworkers reported a more favorable perception of the PSC of their organization. Our study showed that PSC at T1 was associated with lower psychological demands at T2, whereas psychological demands at T2 were associated with increased presenteeism at T3. The data also showed reciprocal relationships between psychological demands and PSC over time. This suggests that teleworkers who experience excessive psychological demands may come to perceive their organization as less supportive of their psychological health and well-being. Our study highlights the interdependence among the organizational context and the perceptions of individuals on their job conditions and, ultimately, their behavior.

### Contributions

This study has several important contributions. Firstly, it responds to a recent call for PSC to be investigated in relation to a broader range of outcomes (Dollard et al., 2019). Most studies on PSC have focused on the core outcomes of the JD-R model such as burnout and engagement. Considering the importance of PSC for outcomes, such as presenteeism, further investigation is needed especially because it is possible to alter PSC through organizational-level interventions (Rickard et al., 2012). The findings lend additional support to JD-R model-based studies having shown the relative contribution of the various task- and organizational-level job resources (Hakanen et al., 2021). Whereas, research has heavily focused on task-level job resources (e.g., skill discretion), our results invite to further examine the specific nature of organizational resources such as PSC within different work settings, including teleworking.

Secondly, the study contributes to our efforts to put the presenteeism behavior in its context. Conceptually, the implications for developing the field are substantial and can add to attempts to offer evidence addressing the observation made by Johns (2010) that the field is theoretical. As argued by Dollard et al. (2019, p. 12): “PSC precedes work quality (such as job demands and resources), it is pronounced as a “cause of the causes” of work stress, and is an upstream theoretical precursor to job design based work stress theories.” Several studies on the antecedents of presenteeism include contextual variables in their predictors, but these are often to combine both variables related to the work environment with variables related to a wider organizational context. Lohaus and Habermann (2019) emphasized the need to distinguish between contextual levels, instead of referring to the context as encompassing all factors that do not refer to the person. Our study brings empirical support for this distinction by considering a contextual distal factor, PSC, and a more proximal factor related to job design, psychological demands. Our results are in line with Liu et al. (2020) who found that PSC positively predicted the perceptions of the working environment, namely organizational support, which in turn reduced presenteeism. Unlike the present study, which uses a longitudinal design with a representative sample of the population of Québec, Liu et al. (2020) use a time-lagged design in which not all variables are measured at each time point, and only with a sample of healthcare workers in China. Although scholars have proposed a person-focused understanding of the presenteeism behavior (Karanika-Murray and Biron, 2020), the presentee cannot be viewed independently of their work environment and organizational behavior cannot be viewed independently of its context (e.g., Grant et al., 2010; Morgeson et al., 2010).

Thirdly, the study is the first to explore the particularities of presenteeism in a sample of teleworkers, since prior to the pandemic, presenteeism was intrinsically linked with physical presence at work. Based on the Health-Performance Framework of presenteeism (Karanika-Murray and Biron, 2020), it is important to consider the relationship of an individual to work and the decision to work or not when ill. Presenteeism can be seen as an adaptive behavior occurring within a complex network of influences in which the worker needs to evaluate the balance resources available to balance the performance demands at work with the health ailment. In the context of telework characterized by social isolation, the absence of formal group norms, and distant leadership, the decision-making process of a worker might depend more on what is more salient in terms of the psychosocial work environment, namely the pressure of psychological demands. This decision-making process has not been investigated in a context where the employee has no physical presence at work and no barriers to prevent working despite illness. As highlighted by Kniffin et al. (2021), the pandemic brought several changes to the workplace, and our study contributes to a better understanding of the potential health-related risks posed by employees being forced into working from home for an extended period.

Finally, using a three-wave design for mediation analysis is uncommon in research on presenteeism. We found the two other studies that have explored the impact of psychosocial constraints on presenteeism using a three-wave design, but neither used a sample of teleworkers nor a contextual higher-level variable such as PSC. In their study, Demerouti et al. (2009) found a reciprocal relationship between burnout and presenteeism over 24 months in a sample of nurses. They showed that, over time, job demands induced pressure to work while ill over time. Similarly, Oshio et al. (2017), using a sample composed of mostly men in the manufacturing sector and a three-wave cohort study, found that job demands were significantly associated with presenteeism 2 years later. Our study brings partial support for a reversed causation between presenteeism and the perception of the PSC as we found that psychological demands at T2 led to a lower perception of the PSC at T3. This points at a downward spiral, in that higher psychological demands, eventually lead workers to perceive their organizational climate in a less positive light, which can lead to increased presenteeism behavior. There is already evidence for this spiral loss as a consequence of presenteeism, where the demands placed on workers take priority over their health, leading them to work while ill, which then leads to further health deterioration (Bergström et al., 2009; Aronsson et al., 2011). A focus on how presenteeism behavior develops over time is needed and can provide new avenues for theorizing in the field.

In terms of methodological contribution, we used a very short (7-day) recall period for presenteeism contrarily to most studies in this field. Presenteeism and absenteeism were both measured as frequencies and in an open-response format. On average, participants reported 1.21 days of presenteeism across each of the three waves, which represents a much higher prevalence compared to pre-pandemic studies and studies that used a 12-month recall period. Only a few studies have used a shorter period of 6 months or less (Lu et al., 2013, 2014; Dhaini et al., 2016; Collins et al., 2018), and all used pre-defined response categories, rendering comparison difficult. In the study of owners and managers, Cocker et al. (2013) used a 4-week recall period and found that 66% of their sample reported at least 1 day of presenteeism (the mean number of days was not reported). Furthermore, as pointed by Ruhle et al. (2019), the temporal order must be consistent, with the antecedents being measured before the predicted variables. Longer recall periods pose threats to the internal validity of the study as the predicted events sometimes occur early in the 12-month period, while the antecedents are measured later. Therefore, also in line with Ruhle et al. (2019), we advocate shorter recall periods to measure the prevalence of presenteeism (e.g., 7 days), using a count measure with an open-response format instead of predefined categories, and with antecedents and consequences. Given that the many changes in health and working conditions were brought about by the pandemic, the context of the study justified a very short recall period.

The rather high prevalence of presenteeism in this study could also be explained by the context of the pandemic. Indeed, we used the definition of presenteeism that included motives related to both physical and mental health. Several studies have shown an increase in mental health problems in connection with the pandemic (Institut National de Santé Publique du Québec, 2020; Salari et al., 2020; Kwong et al., 2021). This increase in mental health problems could explain the high prevalence of presenteeism found in this study. The high prevalence of presenteeism could also be related to the definition of presenteeism we used, namely the behavior of working in a state of ill-health. Following the recommendation by Ruhle et al. (2019), we did not specify a severity threshold (e.g., illness that would justify a sick leave) because, as they argue, this kind of wording implies that the behavior is judged negatively by the presentee. Presenteeism is instead here seen as an adaptive behavior, which serves as a function for the presentee (Karanika-Murray and Biron, 2020). As such, it is neither positive nor negative, it is instead part of a more complex decision-making process. Further studies are needed to investigate the prevalence of presenteeism in the context of teleworking and how preventive interventions can be adapted to their reality.

### Practical Implications

The support we found for the longitudinal effect of PSC on presenteeism *via* psychological demands has important practical implications. Firstly, it shows that, despite the lack of proximity, employers can demonstrate to their employees the importance they place on their psychological health and their commitment to protecting that by adjusting the psychological demands. These results are in line with the practical recommendation by Nordenmark et al. (2019) for organizations to reduce workload and time demands to allow individuals to make the right presence or absence decisions (to work or not to work when ill). A context of high PSC favors balanced job demands, which are aligned with the capacities of workers. The study bolsters calls for top management to make stress and mental health issues a priority.

While COVID-19 has generated many publications in the media on mental health, most of the available resources have focused on self-management tools instead of organizational-level solutions. For example, the Québec government invites people to take care of their health and lifestyle (https://www.quebec.ca/en/health/advice-and-prevention/mental-health#c74786). While these self-help tools are useful and necessary, they put the responsibility on individuals instead of reducing the exposure to adverse work conditions such as excessive psychological demands. This is in line with the recommendation to develop the interventions that are focused more on management practices (Tinline and Cooper, 2019). However, there is evidence showing that managers as a group are also vulnerable and should also be supported during interventions. For example, in a sample of managers, Biron et al. (2018) found that PSC predicted managerial quality during an intervention, but that this relationship was mediated by managers' own level of job control. Managers in that study pointed out that they felt overloaded at work and felt powerless to manage the psychosocial constraints of employees. Their study points toward a cascade effect where PSC improves the psychosocial work environment of managers, which in turn influences their management practices that can have an impact on the health of employees. There are several management challenges associated with the mitigation of the impact of teleworking and place additional constraints on the already heavy workload of managers. We thus argue that interventions should not just target managerial practices, but also include managers as targets for interventions. Enhancing PSC should also involve training and support for strategic decision-makers at the very top of the organization to make mental health a priority.

### Future Research

Future research should aim to understand the aspects of the work context in which presenteeism is situated. Variations in presenteeism climates, norms, and organizational culture, across occupations or sectors are possible. For example, a workplace climate that is competitive and values overtime or in which workers cannot easily be replaced encourages presenteeism (Ferreira et al., 2019). In addition, Aronsson et al. (2000) observed prominent occupational differences in the prevalence of presenteeism. Research on absenteeism behavior has extensively focused on the importance of absence norms and culture (Baker-McClearn et al., 2010). Thus, it is possible that PSC, together with such components of the context, and possibly other aspects of the workplace such as policies and procedures, can together inform a more comprehensive view of the role of workplace climate in the presenteeism behavior.

With the pandemic having revealed an important and weak link in boundary setting, future work on teleworkers and presenteeism can also be expanded in view of the boundary theory (Ashforth et al., 2000) to help understand how weaker or even a lack of physical and temporal boundaries between work and non-work can impact health and performance. Thus, where distinct work–family roles are not possible, or where roles are more blurred, role transitions from one domain to another will be more difficult to delineate among those working from home.

Finally, in addition to further explore the factors linked to the prevalence of presenteeism, it would be useful to incorporate in such longitudinal research, different types of presenteeism that place varying foci on health or performance demands (Karanika-Murray and Biron, 2020). This would help to understand how the workplace and job context shape the balance between health and performance demands and how, over time, they can help to move presentees toward functional presenteeism.

### Limitations

Despite the methodological strengths of the study, its findings should be interpreted considering its limitations. Firstly, we exclusively focused on psychological demands, as opposed to including several mediators such as other types of demands, or resources such as job control, the lack of social support from colleagues or from supervisors, or effort-reward imbalance. Such psychosocial characteristics could play a role in explaining presenteeism and would be consistent with the JD-R model. Our decision to focus on psychological demands was based on the shift in work arrangements that forced workers, from a wide range of occupations and employment sectors, into home-based telework without being prepared for it (Kramer and Kramer, 2020), leading to longer working hours and increased workload (Moss, 2021). This focus on the psychological demands of teleworkers supports the JD-R health impairment process when presenteeism is viewed as an outcome. Future research should identify and investigate the role of other types of job demands for teleworkers. Another limitation concerns the generalizability of the mediation model to other samples (i.e., office-based, or hybrid workers), and to workers from other countries for whom working arrangements are different, which remain to be explored. Despite having a representative sample of the population of Québec in terms of age, gender, and education, data were collected using self-report measures, which are susceptible to self-evaluation bias. Upcoming longitudinal studies should include the data from other sources (e.g., peer perceptions of PSC) and outcomes (e.g., job performance) to increase the scope of the findings. Moreover, our sample of participants showed some differences compared to participants who dropped out, which represents a selection bias. However, as our study focused on those who primarily telework, the observed differences are not surprising as they hinge on the nature of the work itself. Indeed, telework is difficult or impossible for some jobs and employment sectors that are customer-facing, such as transport, trade, food, or tourism. Such a difference was expected given the very nature of telework. Even though the proportion of teleworkers increased dramatically with the pandemic and many people who had never teleworked found themselves working from home, telework still seems to be more common among the more highly educated and among knowledge workers. Finally, regarding the measure of presenteeism, despite the limitations of using a single item to measure presenteeism, this at least allowed us not to measure a phenomenon by its consequence as it is the case when using productivity-based measures of presenteeism (Karanika-Murray and Cooper, 2018).

## Conclusion

Our study shows that despite the distance from the workplace and potential isolation experienced by many teleworkers, the perception that their employer cares about their well-being is important in reducing presenteeism, and psychological demands play a determining role in this process. In their position paper on the “research priorities for the COVID-19 pandemic and beyond,” O'Connor et al. (2020) underline the importance of a better understanding of the impacts of flexible and remote working arrangements on employee mental health and well-being, performance, organizational productivity. While telework can have benefits for well-being (Charalampous et al., 2019) it also has health risks that need to be better understood in light of the organizational context and working conditions.

These results of this research highlight workplace climate as a context to the job (perceptions of psychological demands) and in turn as a context to the presenteeism behavior. Importantly, it has helped to place these influences in a temporal order, thus offering stronger evidence on their causal links. Conceptually, the implications for further developing the field by exploring additional aspects of job design or work characteristics are substantial. Specifically, it would be useful to ascertain which aspects of the PSC are most influential and the mechanism for these influences. For example, How does commitment to mental health by top management or participation and communication to prevent the impact of mental health problems on perceptions of psychological demands? And reversely, How does one's perception of psychological demands support a more positive PSC? It will be worth expanding our investigation into additional aspects of the presenteeism context that work synergistically to impact on the presenteeism decision. Overall, our study provides a more comprehensive framework to conceptualize how an individual, the work, and organizational factors are combined to define presenteeism among teleworkers.

## Data Availability Statement

The raw data supporting the conclusions of this article will be made available by the authors, without undue reservation.

## Ethics Statement

The studies involving human participants were reviewed and approved by Comité d'éthique pluridisciplinaire de l'Université Laval. The patients/participants provided their written informed consent to participate in this study.

## Author Contributions

CB is the principal investigator of this project, conducted the data collection, and performed the statistical analyses with HI. MK-M and CF are co-investigators in this project and contributed to the development of the data collection tools. SS was in charge of the literature review. All authors were involved in the writing process and the revisions.

## Funding

This study was funded by Social Sciences and Humanities Research Council of Canada (SSHRC), Grant 435-2018-0603.

## Conflict of Interest

The authors declare that the research was conducted in the absence of any commercial or financial relationships that could be construed as a potential conflict of interest.

## Publisher's Note

All claims expressed in this article are solely those of the authors and do not necessarily represent those of their affiliated organizations, or those of the publisher, the editors and the reviewers. Any product that may be evaluated in this article, or claim that may be made by its manufacturer, is not guaranteed or endorsed by the publisher.
